# Sleep and brain evolution across the human lifespan: A mutual embrace

**DOI:** 10.3389/fnetp.2022.938012

**Published:** 2022-08-03

**Authors:** Carlotta Mutti, Francesco Misirocchi, Alessandro Zilioli, Francesco Rausa, Silvia Pizzarotti, Marco Spallazzi, Liborio Parrino

**Affiliations:** Department of General and Specialized Medicine, Parma University Hospital, Parma, Italy

**Keywords:** ontogenesis, aging, brain development, sleep, evolution

## Abstract

Sleep can be considered a window to ascertain brain wellness: it dynamically changes with brain maturation and can even indicate the occurrence of concealed pathological processes. Starting from prenatal life, brain and sleep undergo an impressive developmental journey that accompanies human life throughout all its steps. A complex mutual influence rules this fascinating course and cannot be ignored while analysing its evolution. Basic knowledge on the significance and evolution of brain and sleep ontogenesis can improve the clinical understanding of patient’s wellbeing in a more holistic perspective. In this review we summarized the main notions on the intermingled relationship between sleep and brain evolutionary processes across human lifespan, with a focus on sleep microstructure dynamics.

## Introduction

Sleep ontogenesis is a remarkably preserved feature of mammals and has a central role in the human brain development since the very beginning of life.

Solid evidence suggests that sleep maturation and brain morphological dynamics follow similar developmental programs (Frank and Heller, 2003).

Sleep and brain ontogenesis are indeed closely connected, and sleep exerts a pivotal role in brain maturation and plasticity. Three main, non-mutually exclusive, theories have been postulated on the role of sleep in brain development: 1) the ontogenetic hypothesis, according to which REM is a source of endogenous neural activity necessary for brain maturation; 2) the consolidation hypothesis, which defines sleep as a manner of consolidation of experience, emerging especially during the developmental period, and 3) the synaptic homeostasis hypothesis, which emphasises that the main function of sleep is to downscale synapses, while preserving and fortifying a subset. (Frank, 2020).

All three hypotheses agree on the importance of sleep in modulating brain morphology and activity. Accordingly, sleep evaluation, both at behavioural and electroencephalographic (EEG) levels, is diffusely adopted as a non-invasive biomarker of integrative brain development in newborns and children (Dereymaeker et al., 2017). In parallel, sleep anomalies can suggest the presence of underlying processes of neurodegeneration in adult or elderly subjects.

Hence, brain maturation across the lifespan can be largely assessed through sleep dynamics, which indirectly reflect integrity and well-being of neural circuits.

In this review we aim to synthesize the main knowledge on the intermingled relationship between sleep ontogenesis and brain maturation throughout the lifespan, with a focus on sleep microstructure features.

## A glimpse on brain ontogenesis

Brain tissues undergo numerous changes across the lifespan at both cortical and subcortical levels.

Brain morphology is strongly linked to function, with cortical thickness being an indicator of the level of laminar differentiation and cytoarchitecture and, lastly, of the hierarchical position of the specific brain area in the global brain organization (Simic et al., 2005; Wagstyl et al., 2015).

However, brain morphology is far from static, and its volume, shape and thickness dynamically change with age.

First, the development of the brain begins 2 weeks after conception and lasts for more than 20 years. The pre-natal phase is largely determined by genetic factors, while the gene-environment interaction determines the brain development in the post-natal period (Tierney and Nelson, 2009).

During the post-natal phase, malleability of the neocortex is exploited in determining its growth. Individual experiences constitute inputs perceived through the main sense organs, allowing correct brain development; absence of these stimuli leads to an alteration in the organization and function of the central nervous system (CNS), as demonstrated by sensory deprivation influence on the primary visual cortex growing trajectory (Stiles and Jernigan, 2010).

The greatest amount of brain development occurs in the first 2 years, with around 80% of volume growth, rapid myelination of the white matter (WM) and formation of most synapses (Huang et al., 2015).

Human total brain volume increases by about 0.5% annually until adolescence and then declines by around 0.2% per year during adulthood, along with a concomitant increase in ventricles volume (Narvacan et al., 2017).

The final brain size is at least partly pre-determined thanks to a highly complex genetic–epigenetic interaction, and may be seen as a continuum with abnormal values as the extremes. Notably, elegant genome wide associations studies have pointed out how total brain volume and intelligence might share the same genetic factors (Jansen et al., 2020).

Regional changes in brain volumes can be heterogeneous: for example, if grey matter volumes of the frontal, parietal and occipital lobes follow a linear decrease with aging (Allen et al., 2005), the hippocampus volume presents a curvilinear trend, with largest declines occurring after the age of 60 (Beheshti et al., 2019). The major age-related changes in grey matter (GM) volumes can be observed over the frontomedial, insular, inferior frontal gyrus and inferior parietal areas, while the middle and inferior temporal gyrus and the occipital brain regions are partially spared (Ziegler et al., 2012).

The volume of all subcortical structures, except the caudate and globus pallidus, linearly decreases with aging (Brabec et al., 2003; Narvacan et al., 2017), following an intra- and interhemispherical dependency (Gomez-Ramirez and Gonzalez-Rosa, 2022).

Meanwhile, WM tracts undergo enormous variations along the aging process, which interestingly do not overlap with GM dynamics. On average, it is estimated that around 25%–45% of WM changes during the lifespan are modulated by age (Yeatman et al., 2014).

At the macrostructural level, WM follows a nonlinear relationship (inverted U-shape) within the age range 20–86 years, best fitting a quadratic model: in other words, WM values increase with age, reaching a peak around the fourth decade followed by a significant drop thereafter (Matsuda, 2013). More in detail, maturation differences occur also at the level of the distinct tissue types which compose the WM (astrocytes, microglia, myelin...), each of them apparently following their own evolutionary process (Yeatman et al., 2014). Consequently, a single model to predict all the adult variations in WM microstructure cannot be exhaustive.

Diffusion tensor imaging (DTI) studies show a gradual decrease of fractional anisotropy, a measure for fibers density and microstructure integrity, after the age of 30, with a subsequent steeper decline after the age of 50. Conversely, mean diffusivity and radial diffusivity, indicators of the overall magnitude of water diffusion related to size/density of axon bundles, myelin lipid contents and number of cells, initially decrease until 40 years and then gradually increase (Beck et al., 2021).

Notably, WM changes observed with aging do not “revert back” to the biology of a child’s brain and are not always symmetric in the two hemispheres, being probably influenced by numerous endogenous and exogenous processes and by acquired lifetime experiences.

If brain volume and its progressive decline over time are partially pre-determined, principally thanks to genetic and biological factors, neuronal plasticity is set to be a fascinating manner for maintaining homeostasis and determining a healthy aging. The concept of cognitive reserve is closely connected. Recently, several studies have focused on its dynamics, inquiring on the possible formation of new neurons and synapses in the hippocampus and in peri-ventricular areas in adulthood, being still debated the chance of a global neurogenesis in adult human brain (Van Loenhoud et al., 2018). Unlike neuronal population, glial progenitors (mainly oligodendrocyte progenitor cells) can instead migrate and proliferate even in adulthood, as it occurs in response to traumas.

The dynamic variations in brain morphology are accompanied by relevant functional adaptive processes, which can be revealed thanks to functional imaging techniques. Fluorine-18-fluorodeoxyglucose positron emission tomography (F-FDG PET) studies show how brain metabolism with aging decreases in both supra and infratentorial regions, sparing the cerebellum, with greater reduction in both frontal lobes (up to 40% of glucose metabolism reduction in healthy old people), with interesting gender differences (Hu et al., 2013; Pourhassan Shamchi et al., 2018).

## Brain networks and sleep

The brain can be depicted as a complex multi-layer system made up of highly interconnected regions, which is frequently deemed as “connectome,” During the first months after birth, brain functional connectivity is stronger within local networks, while, by the end of the first postnatal year, brain connectivity better supports the “longer distance” connections (e.g., frontal to visual and frontal to sensorimotor regions), with the exception of the posterior cingulate cortex which maintains a high level of intra-network connectivity profile (Damaraju et al., 2014). Thereafter, a progressive linear decline of functional connectivity within the major cortical networks occurs with aging, e.g., default mode network, sensory-motor network, auditory network (Liu et al., 2017).

Analysing brain connectivity throughout the lifespan,Puxeddu et al. (2020) found that brain connectivity patterns follow a U-shape curve, characterized by a strong within-modules connectivity in the first years and by a robust inter-connectivity across modules in the late lifespan. Brain modules tend to reconfigure with aging, suggesting a progressive gain in terms of brain flexibility, but they also gradually loose numerous inter-hemispheric connections, probably due to shrinking of the corpus callosum.

Interestingly, sleep is essential for neuronal plasticity. Processes of synaptic reorganization-renormalization take place especially during sleep and a topographic correlation has been found between synaptic modifications occurring in wakefulness and slow wave activity (SWA) onset in the following sleep (Ringli and Huber, 2011; Gorgoni et al., 2013). Moreover, sleep disturbances may alter the proper course of brain ontogenesis. In rats, sleep deprivation has been shown to hamper the normal neurogenesis and cell proliferation (Guzman Marin et al., 2003). Recent studies in healthy children have shown how a lower amount of sleep leads to a bilateral hippocampal volume reduction (Taki et al., 2012). The impact of sleep disorders in promoting a greater age-related brain atrophy and a steeper cognitive decline in older adults is a well-known issue, lastly favouring the development of neurogenerative diseases (Lo et al., 2014; Sun et al., 2020). Further investigation has also focused on the bidirectional relationship between sleep and altered brain aging: increased neuronal activity and clearance mechanism alteration, that accompany numerous neurodegenerative disorders may lead to the accumulation of a misfolded protein (as occurs in Alzheimer disease), which, in turn, damages various brain areas, such as the suprachiasmatic nucleus, a pivotal structure for sleep and circadian rhythms regulation (Nassan and Videnovic, 2022).

## Sleep evolution across lifespan

Sleep could be considered a behavioural state, with all vertebrates and many invertebrates presenting some form of sleep. Comparative studies highlighted how almost all mammalians and birds exhibit an alternating cycle of slow waves sleep (SWS), interrupted by episodes of rapid eye movements. Moreover, in the animal kingdom sleep deprivation is a debt that needs to be paid. The need of being vigilant for predators or the necessity of surface breathing makes it almost impossible to sleep continuously for hours. Several species have consequentially overcome this conflict by engaging in un-empispheric SWS (a peculiar mono-hemispheric sleep state) (Lee-Chiong, 2005; Keene and Duboue, 2018).

Sleep development requires the integration of numerous brain networks both at cortical and subcortical level. In parallel, brain rhythms change and evolve, mainly during sleep, with great variations in the first period after birth.

Newborns spend most of their time asleep; however their sleep structure is largely different from the adult one. Starting from the third trimester of pregnancy four developmental brain states can be identified including active sleep (AS), quiet sleep (QS), active wakefulness and quiet wakefulness (Bourel-Ponchel et al., 2021).

The last weeks of gestation in the third trimester are characterized by an absolute dominance of AS, which parallels the rapid brain evolution of this age and the early maturation of brainstem areas, key factors in the AS build-up (Mirmiran et al., 2003; Valtax et al., 2004).

AS is a precursor of REM sleep with atonia still undeveloped, it usually appears immediately or soon after sleep onset, and persists, with various duration, during the whole sleep period (Roffwarg et al., 1966).

Interestingly, in animals fully developed by birth, REM sleep declines rapidly, while in animals (and humans) who undergo an important increase of brain networks postnatally, REM sleep persists for longer periods and thereafter gradually diminishes in percentage, in favour of NREM sleep (Jouvet-Mounier et al., 1970).

Through its distributed neural activation, AS (and then REM sleep) can provide a high level of endogenous stimulation in epochs where foetuses or newborns have limited access to environmental stimuli (Mirmiran et al., 2003). Conversely, when neonates are finally exposed to multiple external stimulations, NREM sleep emerges as the dominant stage.

The main EEG feature of AS is the presence of synchronised delta activity, interrupted by theta oscillations. It has been recently postulated that these theta oscillations could promote the synchronization of different brain structures, building an easy road for cortical and subcortical networks development. Del Rio-Bermudez and Blumberg (2018) have shown how in newborn rats oscillations during AS, especially if twitch-related, promote neuronal synchrony across distant sensorimotor structures and cortico-hippocampal communications.

Other theories focus on the significance of REM sleep on the consolidation of novel memories (Walker and Stickgold, 2004) and on the preparation of highly reactive adrenergic receptors for waking periods, compared to the more “restorative” NREM sleep (Zepelin and Rechtschaffen, 1974).

REM-deprivation in animals hamper cortex maturation and brain plasticity development, limiting the adaptive brain remodelling which is physiologically induced by animals’ exposure to enriched environments (Mirmiran et al., 1983). Furthermore, Shaffery et al. (2006) showed how REM-sleep deprivation in adolescent rats could alter the balance between excitatory and inhibitory mechanisms in the visual cortex, suggesting how, despite its relative decline with aging, REM-sleep may continue to play an important role beyond mammals’ childhood.

Between four to 6 months after birth, AS evolves into REM sleep and QS becomes NREM sleep, with further subdivision into stages N1, N2, and N3 (Anders et al., 1995).

The development of such an organized and predictable pattern of sleep states alternation requires the stabilization of afferent connections with deep nuclei such as thalamus, hypothalamus and hippocampus. For a detailed review of prenatal cerebral connections development see Kostovic and Jovanov-Milosevic (2006).

Schematically, starting from 20–23 gestational weeks the foetus develops the first thalamo-cortical connections, mainly involving primary sensory cortical areas; afterwards, rough synapses take shape. Gradually, cortico-cortical connections and axonal arborisation progressively increase. Corpus callosum undergoes an enormous morphological evolution, increasing its dimension to a maximum, and a huge number of cortical synapses can be identified, mostly within the 4^th^ cortical level. The shorter cortico-cortical connections mostly take place after birth, together with an intensive synaptogenesis, under the stimulation of new experiences. Lastly, the gradual restoration of an optimal level of cortical connections occur, guarding against the synaptic overload (Kostovic and Jovanov-Milosevic (2006)).

Traditionally, the REM sleep regulation has been strongly linked to the brainstem region. Specifically, the caudal laterodorsal tegmental nucleus (LDT), sublaterodorsal nucleus (SLD) and precoeruleus region are considered the main REM-regulatory elements. REM-on glutamatergic neurons of the ventral SLD mediate REM motor atonia through direct synaptic activation of glycinergic interneurons of the spinal ventral horn as well as *via* GABAergic/glycinergic neurons of the ventral medial medulla. Lateral hypothalamic orexinergic neurons provide excitatory control on the LPT (lateral pontine tegmentum) neurons. Cholinergic laterodorsal tegmental and pedunculopontine tegmental (LDT/PPT) neurons may produce REM sleep through activation of REM-on SLD neurons. Finally, lateral hypothalamic neurons containing melanin-concentrating hormone contribute in the REM sleep regulation, possibly through the direct inhibition of REM-off ventrolateral periaqueductal grey/LPT neurons (Fuller et al., 2007).

Since Von Economo’s research on Lethargic Encephalitis, the hypothalamus has been considered a central hub in the sleep/wake regulatory pattern (Von Economo, 1930). It is diffusely accepted that the ventrolateral preoptic area and the median preoptic nucleus contain neurons essential for promoting and maintaining non-REM sleep (Hobson et al., 1975). The role of the hypothalamus on REM sleep is instead a quite recent discovery. New evidence suggests the existence of an extra-pontine REM regulation, strongly linked to REM active hypothalamus neurons (Bjorkum et al., 2002).

Vice versa, NREM sleep regulation, which has been historically linked to the inhibitory activity of the ventro-lateral pre-optic area (VLPO) on the wake-promoting brain areas, seems to have one of its main neural substrates at the cortical level, where a population of neurons releasing GABA and nitric oxide (NO) capable of the wide-spreading increase of slow waves can be found. These cells produce NO in response to sleep pressure and homeostatic drive, favouring the long-range synchronization pattern that sustains the appearance of slow waves (Scammell et al., 2017). Given the complexity of brain circuits involved in sleep kinetics, it is not entirely surprising that it takes time for neonates to fully develop adult-type sleep-wake cycles.

Moving into circadian dynamics, neonates present a well-defined sleep cycle with a short ultradian cycle of around 30–70 min (Scher et al., 1992), and full-term neonates can organize their sleep cycles over multiple internal and external cues (e.g., light/dark cycle, temperature, noise, environmental stimuli and interactions), which sustain the synchronization of their internal clocks.

Neonates spend as much as 80% of their days asleep, waking mostly for feeding necessities (Mindell et al., 1999). Afterwards, due to their increased capability to absorb calories, essential for growing, they reach a more continuous sleep period during the night and globally ameliorate their sleep efficiency (for the joy of parents!) (Blunden and Galland, 2014).

With the transition into childhood, there is a progressive decline of total sleep time, wake-after-sleep-onset, stage N3, REM sleep duration and global number of sleep cycles per night. In parallel, sleep undergoes an improvement of sleep efficiency, with an increase of mean cycle duration and number of stage shifts and stage N2 percentage (Scholle et al., 2011). Instead of sleeping randomly, children learn to concentrate their sleep period during the night, with the persistence of brief daily naps.

Mean sleep duration changes from 12.8 h (infants) to 11.7 h (toddlers, 12–35 months) and 10.4 h for pre-school children (3–5 years-old) (Sleep foundation, 2022).

The paired suprachiasmatic nuclei (SCN), located above the optic chiasm in the anterior hypothalamus, are a biological clock, the neuroanatomical basis for circadian rhythms’ genesis. Animal studies have shown how this clock is fully formed and begins its characteristic oscillatory pattern even *in utero*, but, although monkeys and humans share numerous ontogenetic similarities, the time needed to achieve a stable and continuous circadian rhythm is much longer in humans than in other mammals (Van Eerdenburg and Rakic, 1994; Weaver, 1998). This feature might be due to a higher level of complexity in human chronobiology, but could be partly motivated by a harder synchronization between external stimuli (attention of caregivers, environmental interactions) and endogenous rhythmicity (Rivkees, 2003).

Adolescence is a developmental period dominated by huge hormonal changes: the adolescent’s brain undergoes structural variations in terms of brain morphology, with GM thickness and surface area gradually decreasing, showing a greater impact over the frontal area (particularly the orbitofrontal cortex and the anterior cingulate cortex), while WM volume increases over time (Herting and Sowell, 2017; Vijayakumar et al., 2018). Numerous studies focused on their ontogenetic changes during adolescence, with a special focus over the hippocampus, nucleus accumbens, pituitary gland and amygdala. As for animals, it seems that much of the volumetric changes in humans might be related to sex hormones receptors (Vijayakumar et al., 2018). Although there are still some inconsistencies regarding gender-related differences in terms of brain dynamics and with respect to hormonal changes however it seems that greater puberal development and/or higher testosterone levels are associated with stronger morphological variations (Vijayakumar et al., 2018).

In parallel to the described variations in terms of brain morphology, at the EEG level various changes take place in terms of frequency, with a steep decline of the theta power (4–8 Hz) and the delta power (1–4 Hz) starting respectively around 9 and 11 years (Feinberg and Campbell, 2010). The decline in delta power has been linked with the cortical synaptic pruning process: during adolescence the excess of synaptic connections probably decreases the number of neurons able to fire in synchrony, lastly reducing slow waves amplitude (Campbell et al., 2012). Accordingly, the decline in the delta power activity follows a specific trajectory moving from back-to-front cortical areas, in parallel with cortical thinning and maturation (Gogtay et al., 2004; Kurth et al., 2010). In particular, the theta power curve parallels the earlier thinning of the 3-layer allocortex, which can be detected with MRI investigation, while the delta curve follows the maturation of the more complex and dynamic 5-layer isocortex, starting from the frontal areas (Campbell and Feinberg, 2009).

In this framework, cortical slow waves somehow mirror the GM changes. The dominance of SWA during childhood might reflect the increase of synaptic density typical of this age and the need for a recruitment of a wider sample of neurons/larger networks to perform tasks (Buchmann et al., 2011a).

Female adolescents exhibit greater sigma power, spindle density and amplitude during NREM sleep, which has been linked to cognitive performances (Bodizs et al., 2014; Ricci et al., 2021). Likewise, females present greater power at higher frequencies (beta and gamma) during both NREM and REM sleep periods, a difference whose anatomical counterpart is still controversial (Markovic et al., 2020) and differ from men in terms of sleep EEG coherence, an indirect measure of functional connectivity, which results higher in females across most frequency bands (Lenroot et al., 2007; Markovic et al., 2020).

Notably, an impressive number of adolescents complain pf sleep difficulties and shorten their sleep periods (Bruce et al., 2017). This can be due to their tendency to postpone the timing of sleep onset, mostly for social and hormonal reasons. The reduced sleep quality in adolescence might negatively impact wellbeing and health from multiple perspectives including a higher risk for obesity, an enhanced pain perception, an increased risk for depression and healthy-risk behaviours.

Thereafter, sleep continues to change during adulthood: sleep duration, sleep efficiency, percentage of stage N3 and REM sleep decrease with age, whilst stage N1, stage N2 and sleep latency slightly increase (Ohayon et al., 2004).

According to a recently published systematic review with meta-analysis, in adults every 10 years there is a decrease of total sleep time by 10 min, of sleep efficiency by 2%, of wake after sleep onset increase by 10 min, with an increase of sleep onset latency by around 1 min and of arousal index by two events per hour (Boulos et al., 2019).

Biochemically, throughout the night, adult sleep is modulated by distinct neurotransmitters: to simplify the sleeping brain can be depicted as a train composed of five wagons, each lasting 90 min and representing a complete sleep cycle. The strating three carriages, collected in the first 4.5 h of the night, are sustained by the gamma-aminobutyric acid (GABA) and support deep sleep propensity, whilst the last two wagons are linked to activating neurotransmitters including acetylcholine, and prepare the brain to morning awakening (Vanini et al., 2012; Jones, 2020) This biochemical difference partly explains why sleep becomes frailest as we move to the early morning hours.

Changes in the sleeping brain rhythm develop in a topographically organized direction with delta declines being major over medial cortical areas and sigma decline over fronto/central regions. Once again, these variations reflect the ongoing dynamics of GM and WM impoverishment, which paraphysiologically accompany the aging process (Fitzroy et al., 2022).

Although the reduction of slow frequency among elderly has been associated with cortical thinning in the same brain areas (e.g., reduction of delta power over frontal cortex associated to cortical thinning in the frontal areas), recent evidence suggests that the relationship between rhythms and brain morphology is less clear-cut. Indeed, delta activity reduction might reflect the progressive thinning over temporal and occipital cortices (including limbic and paralimbic brain areas), which are extensively connected with frontal areas. Hence, the degeneration of frontal cortices negatively influences the downstream brain regions which play a key role in the delta generation (Fitzroy et al., 2022).

Moving deeper into the same topic, previous studies explored the dynamics of EEG power spectrum and sample entropy between adults and elderly, with some interesting results (Bruce et al., 2009). In particular, sample entropy, a measure for networks interactions complexity, was found to be higher in elderly subjects (mean age 78 ± 2 years) during stages N2 and REM, suggesting a higher predisposition towards a more “awake” cortical state and, indirectly, a decrease of their sleep “attitude.” In the same perspective, decline in delta power is combined with an increasing higher frequency of beta power, an indicator of cortical arousal (Carrier et al., 2001). In other words, the change in the neural entropy balance induces a decline of the sleep-promoting forces in favour of a more vulnerable sleep period.

Finally, older adults are especially sensitive to sleep disturbances, due to the combination of age-related changes in sleep architecture and a higher rate of sleep disorders, such as insomnia and obstructive sleep apnea syndrome (OSAS). Though a detailed description of sleep disturbances in older people is beyond the scope of this review, it is widely accepted that the presence of OSAS is associated with poorer cognitive performances and sleep disruption beginning in midlife is predictive for the development of dementia in later life (Yaffe et al., 2014) (Bah et al., 2019).

## Sleep microstructure dynamics across lifespan

Sleep complexity extends widely beyond its macrostructural features, and numerous oscillatory activities can be detected at macro-, meso- and microscale levels during human sleep.

At the mesoscale level, periodic activities can be identified, deeply embedded within sleep structure. NREM sleep is enriched by K-complexes, spindles and periodic electrical activities such as the cyclic alternating pattern (CAP). These elements are representative of the dynamic dialogue between thalamus and cortex and strongly correlate with the regulation of vigilance suppression/sleep preservation and memory consolidation (Ioannides et al., 2017; Niethard et al., 2018).

**FIGURE 1:**
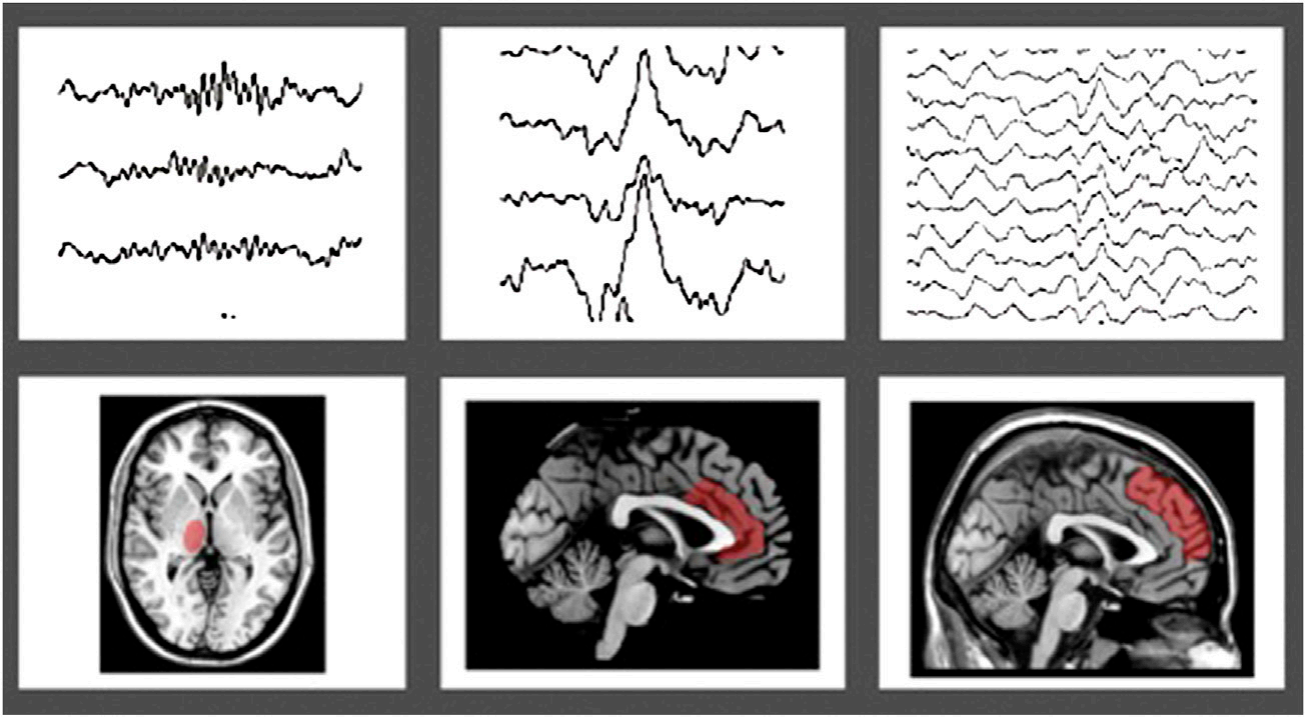
Topographic projection of sleep figures in the human brain, highlighted in red: panel **(A)** sleep spindles and thalamus; panel **(B)** K-complex and anterior cingulate cortex; panel **(C)** Slow waves sleep (SWS) and frontal gyri.

In details, K-complexes are high-amplitude biphasic waves, representing a cortical “down-state” generated in widespread cortical areas, with a maximum representation over the frontal cortex, which can either contribute to sleep fragmentation or rather lead to sleep reinforcement (Mak-McCully et al., 2015; Halasz, 2016), as Janus-face sleep-elements, including an initial arousal followed by a sleep protective counterpart (Kolja Jahnke et al., 2012). They can either appear spontaneously or be evoked by external stimuli (e.g., noise) and probably reflect the activity of purely “cortically-generated” slow oscillations, rather than a thalamo-cortical connection (Steriade et al., 1993). Recently, the anterior cingulate cortex has been suggested to be one of the K-complex generators (Ioannides et al., 2019) (Figure 1 **)**.

Aging imposes a steep decline in the evoked K-complexes amplitude and density, with a maximum over frontal regions, suggesting that these sleep elements might act as an indirect measure of how aging impacts the functional integrity of brain networks (Colrain et al., 2010; De Gennaro et al., 2017). The reduction of both K-complexes and slow ave sleep (SWS) seems to be linked to the paraphysiological drop of the homeostatic pressure regulation among the elderly (Dorffner, et al., 2015).

Spindles are wax-and-waning electrical activities generated in the thalamus and spreading to the fronto-central cortex using thalamo-cortical projections. Spindles create an electrical background particularly favourable to brain plasticity, as they mediate a temporary unresponsiveness to external stimuli, preventing sleep-dependent mechanisms from interference. Growing evidence highlights the existence of two types of sleep spindles: the slow ones (<12 Hz) and the fast ones (>12 Hz); only the first are deeply associated with cortical slow-oscillations up-states, exerting a clear modulatory role in the sleep-related learning processes (Molle et al., 2011). On the other hand, fast spindles are strongly synchronized with hippocampal sharp-waves ripples and tend to manifest during cortical up-state of slow oscillations during NREM sleep and sustain hippocampal-dependent sleep learning processes (Azimi et al., 2021).

Whether spindles may be considered as a global phenomenon rather than a local process is still debated, as these EEG phasic events may occur with various frequency and no/partial coherence between distinct brain areas (Nir et al., 2011).

Some similarities can be identified in premature neonates between delta brushes and sleep spindles as they both share similar topography (starting with a central distribution) and frequencies. Hence, they both have been linked to brain plasticity (Clawson et al., 2016). Mature sleep spindles can be detected around 3 months after birth, in the central brain cortices, thereafter they continue to increase in terms of coherence and move anteriorly to the fronto-temporal areas (Hagne, 1972; Louis et al., 1992).

Sleepspindles progressively decline with aging, with a decrease in terms of density that is more pronounced over frontal and occipital sites (Martin et al., 2013) and, during adulthood, a greater sleep spindle density can be assessed in women, suggesting functional gender-dependent differences in the thalamocortical circuits (Huupponen et al., 2002).

CAP is considered the neurophysiological hallmark for sleep instability and one of the major guardians of sleep resilience (Parrino and Vaudano, 2018). CAP oscillations are organized in sequences each one composed of a phase A (activation or “greater arousal”) and a following phase B (de-activation or “lesser arousal”) (Terzano et al., 2001).

Phase A can be further subdivided into three subtypes, based on their composition in terms of slow waves and fast rhythms: subtype A1 (high amplitude slow wave >50% of the entire phase A duration), A2 (balanced mixture of slow and fast rhythms) and A3 (>50% of rapid low-voltage activities).

**FIGURE 2:**
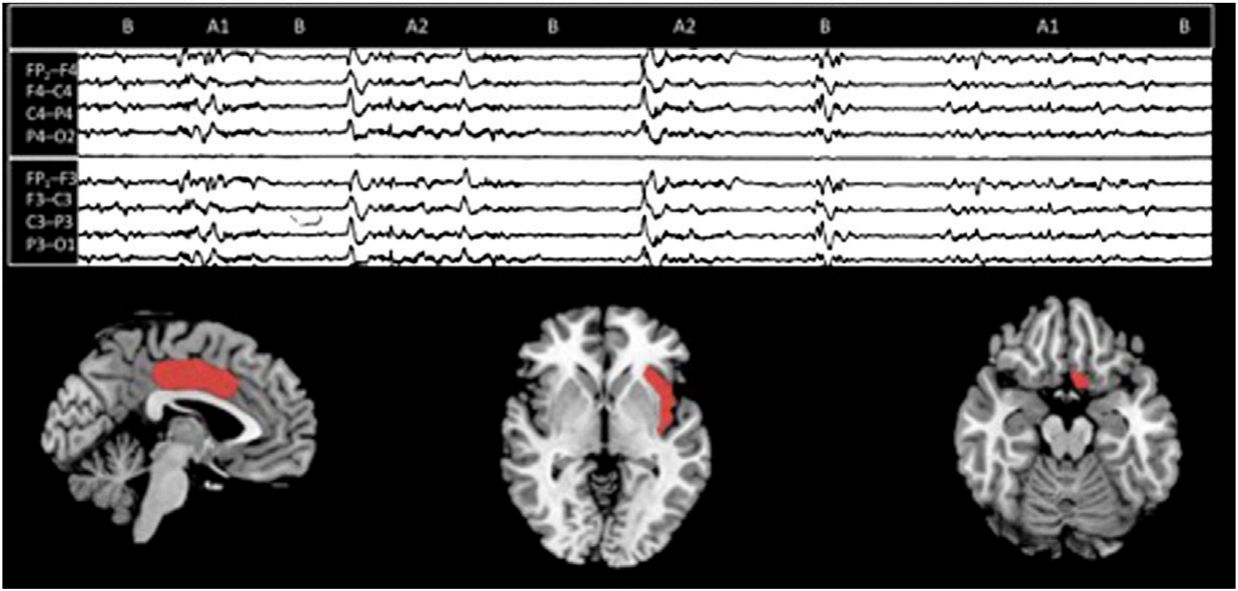
On the top, a CAP sequence (composed of A and B phases). On the bottom, according to a stereo EEG—fMRI study, the three suggested CAP brain sources [from the left: middle cingulate gyrus (Brodmann area 24), insula (Brodmann area 14/15), and the basal forebrain [Brodmann area 25]] (modified from Kokkinos et al., 2019).

Although data regarding CAP topography are still fragmentary, various source investigations confirmed the prevalence of subtypes A1 over the anterior brain areas, whilst A2 and A3 dominate over the parieto-occipital lobes (Ferri et al., 2005; Kokkinos et al., 2019) (Figure 2).

The most “practical” measure to evaluate sleep microstructure is the CAP rate (percentage of NREM sleep occupied by CAP sequences). According to a systematic review, CAP rate gradually increases with age, with a U-shape pattern, being higher in the first years after birth, with a significant drop during middle age and a final increase during advanced adulthood (Parrino et al., 1998). However, some important differences should be underlined: CAP in infants and toddlers is mainly constituted by subtypes A1, while it appears largely associated to a high proportion of CAP subtypes A2 and A3 in the elderly (Bruni et al., 2010; Migueis et al., 2021). This difference is not trivial as CAP subtypes present distinct roles in terms of sleep/wake organization.

CAP subtypes A1, electrophysiologically characterized by a high proportion of slow waves, parallel the homeostatic process, and sustains the build-up of deepest sleep stages. Conversely, the more disturbing CAP subtypes A2 and A3, recognizable by their richness in low-amplitude fast rhythm, appear strongly linked to ultradian cyclicity, anticipating REM sleep and infra-sleep awakening, and typically prepare the brain to lighten sleep (Terzano et al., 2005; Halasz et al., 2014).

CAP subtypes A1 have been linked to sleep-dependent learning processes (Ferri et al., 2008), while the excess of subtypes A2 and A3, typical of various sleep disorders such as OSAS (Gnoni et al., 2021), may hamper sleep-associated cognitive improvement.

Neonates present a typical EEG pattern, the Tracé Alternant (TA), which was initially considered as the main precursor of CAP. The TA is a transitional pattern of QS characterized by alternating periods of short-duration, high-voltage activity (bursts) separated by lower-voltage activity (inter-bursts). Although it may seem somehow similar to CAP, it has been demonstrated that the two processes do not share the same significance in terms of sleep physiology. TA disappears when more stable cortico/subcortical connections mature and a more recognizable NREM sleep develops. In this scenario, a physiological maturation of the EEG is crucial for CAP appearance (Miano et al., 2009).

Following the SWS decline that physiologically occurs with aging, CAP undergoes a similar trend, with relevant difference in the low frequency (<7 Hz) range power spectrum between children and adults, especially during sleep stages N2 and N3 (Bruni et al., 2009).

Unfortunately, most works on CAP focus on pediatric and adult populations, while conclusive normative data in elderly sleepers is limited. However, recent investigation suggests that CAP impoverishment might be related with neurodegenerative processes (Maestri et al., 2015; Carnicelli et al., 2019; Melpignano et al., 2019). Similarly, the age-related decrease of SWS, morphologically associated with prefrontal GM atrophy, and of sleep spindles, appear linearly correlated with impaired long-term memory in the elderly.

Thus, sleep microstructure features should be reasonably included among the leading pathways of aging-associated memory loss and their evaluation should be adopted to assess brain maturation, mirroring the development of healthy neural connections and adequate aging processes. (Mander et al., 2013).

## The unavoidable link between brain anatomy and sleep physiology

Despite the growing knowledge on the dynamic of brain and sleep maturation, the two processes are rarely considered in an inclusive perspective.

Individual features in terms of sleep dynamics might–at least partly- reflect underlying brain anatomical differences. It has been demonstrated that individual EEG power spectra during sleep are largely preserved through lifespan, supporting the concept of their fingerprint-like significance (Eggert et al., 2022). This uniqueness may influence the trajectory of brain development modulating vulnerability and robustness towards internal and external stressors. Structural disparities in white inter-hemispheric tracts can influence the “variability” of NREM SWA, supporting the role of long white matter fibres in the synchronization of neural activity (Buchmann et al., 2011b). Reasonably, these tracts and particularly the corpus callosum, can promote the achievement of a more coherent firing pattern between coupled brain areas during sleep, thus assisting the development of synchronized slow waves. The relationship between sleep dynamics and grey matter evolution is less clear, due to inconsistency of published results (Paulekiene et al., 2022).

As already outlined, sleep can inform over brain tissue integrity also at the microstructure level: indeed CAP can be used as non-invasive biomarker to predict severe neurodegenerative processes (Maestri et al., 2015; Melpignano et al., 2019).

Hence, morphologically an intact brain is required to sustain healthy sleep dynamics. On the other side of the coin, disruption of the sleeping brain harmony may jeopardize the maintenance of a regular brain tissue, as confirmed by several studies highlighting the association between poor sleep and dangerous brain consequences, with accelerated brain aging (Finelli et al., 2001; Ramduny et al., 2022).

Therefore, the unavoidable “mechanicistic” link between sleep, expressed as an ancestral human function, and brain morphology, is remarkably tight and requires attention from a clinical perspective. This point, however, has been largely neglected so far, as confirmed by the lack of neuroradiological confirmation for any sleep disorder diagnosis, according to current international guidelines. Although it could seem contradictory to investigate sleep–mainly an electrophysiological and behavioral phenomenon—adopting a neuroradiological techinique such as brain MRI, this ambiguous scenario has already been tackled in various neurological conditions with similar background such as epilepsy, confirming the inextricable link between electrophysiology and morphology (Concha et al., 2012; Liu et al., 2016).

In summary, given the bidirectional relationship, the characterization of brain anatomy with cerebral MRI, ideally integrated with advanced neuroimaging methods to overcome the naked eyes performance, may be useful to integrate the evaluation of patients affected by sleep disorders, identifying individuals’ frailty and pointing out subjects warranting a higher level of concern. In parallel, an accurate sleep history strongly supports the general clinical vignette and can integrate the radiologist’s understanding of health and wellbeing.

## Conclusion

Human brain plasticity is a puzzling topic which is far from being fully understood. Since the very beginning of life, the brain is never the same, shifting throw a multitude of changes in several timescales.

Sleep is even a bigger enigma. Breaking it down in its simpler elements, mainly exploiting the EEG technique, we can measure and categorize its structure and evolution. However, conventional measures approach, which are essential from a clinical perspective, may underestimate its dynamics and complexity.

The present review summarizes the main knowledges on sleep and brain maturation throughout the lifespan and shows how brain and sleep changes run in parallel.

Using morphological modifications associated to sleep changes, we can better define the origin of nocturnal rhythms. Similarly, we can adopt sleep macro- and micro architecture as a non-invasive biomarkers of brain integrity.

Sleep and brain morphology are causally inter-connected, and the disruption of one impairs the other (Table 1 **)**. As mentioned above, age-related frontal cortical thinning leads to a reduction of K-complexes and A1 CAP phase density. Meanwhile, as recently shown, even a single sleepless night strongly impacts brain morphology, determining several brain volumes shifts (Jansen et al., 2020).

**TABLE 1:** Schematic synthesis of the most noticeable sleep changes across lifespan.

Sleep dynamics across lifespan	Perinatal	Childhood	Adulthood	Elderly
Sleep macrostructure	AS evolves into REM sleep and QS evolves in NREM sleep (0–4/6 months)	Decline of TST/WASO/SWS/REM sleep stage/number of sleep cycles	Decrease in sleep duration, SE, SWS and REM sleep stages	Higher sample entropy in NREM N2 and REM sleep stages
	Short ultradian cycle (30–70 min)	Increase of 1) mean cycle duration; 2) number of stage shift,; 3) N2 stage percentage	Increase in light sleep stages of NREM sleep; increase of SE	Decrease of SE/higher rate of sleeps disorders (e.g., OSA and insomnia)
	Mean sleep duration: 12.8 h	Mean sleep duration: 11.7 (toddler) 10.4 (3–5 years old)	Every 10 years reduction of total sleep time by 10 min and sleep efficiency by 1%–2%	
Sleep microstructure	Alternating periods of short-duration, high-voltage activity separated by lower-voltage activity (TA); appearance of sleep spindles (3 months); High levels of CAP fluctuations (mainly subtypes A1)	Increase density and coherence of sleep spindles; persistence of high levels of CAP oscillations	Gradual increase of CAP rate (subtypes A2 and A3; decrease of subtypes A1)	Decline in K-complex and spindles amplitude and density; Steeper increase of CAP rate (subtypes A2 and A3; decrease of subtypes A1)

AS, active sleep; QS, quiet sleep; TST, total sleep time; WASO, wake after sleep onset; SWS, slow wave sleep; SE, sleep efficiency; OSA, obstructive sleep apnea; TA, trace alternant; CAP, Cyclic alternating pattern.

There is an inextricable and dynamic relationship between sleep and brain morphology. Sleep clinicians should always be aware of the complexity underlying cerebral rhythms during sleep, as it can lead to a deeper understanding of brain functioning and maturation.
